# HOPE or hopeless? Unexpected neurological recovery after ECPR rewarming in a patient with severely hypothermic cardiac arrest with multiple haemorrhagic complications: a case report

**DOI:** 10.1186/s13049-025-01497-x

**Published:** 2025-11-20

**Authors:** Tatsunori Nagamura, Yuya Yoshimura, Sho Terashige, Tetsuro Kiyozumi

**Affiliations:** 1 Department of Traumatology and Critical Care Medicine, National Defense Medical College Hospital, 3-2 Namiki, Tokorozawa, Saitama, 359-8513 Japan; 2 Department of Emergency and Critical Care Medicine, Hachinohe City Hospital, Aomori, Japan; 3 Department of Orthopedics, Hamawaki Orthopaedic Hospital, Hiroshima, Japan

**Keywords:** Hypothermic cardiac arrest, Extracorporeal cardiopulmonary resuscitation, Veno-arterial extracorporeal membrane oxygenation, Subdural haematoma, Cardiac tamponade

## Abstract

**Background:**

A high Hypothermia Outcome Prediction after Extracorporeal Life Support (HOPE) score may indicate favourable neurological outcomes in hypothermic cardiac arrest, even among elderly adults. Although the score does not account for life-threatening complications arising during hospitalisation, outcomes may nevertheless align with its favourable estimates.

**Case presentation:**

A 90-year-old woman with a history of hypertension and mild dementia was found collapsed in a cold environment. On arrival, her core temperature was 22.0 ℃, and she developed ventricular fibrillation during transport. After 90 min of cardiac arrest, veno-arterial extracorporeal membrane oxygenation (VA-ECMO) was initiated. Her HOPE-estimated survival probability was 76%. Shortly after admission to the intensive care unit, cardiac tamponade secondary to myocardial contusion was suspected and managed conservatively with pericardial drainage and massive transfusion. Following ECMO weaning on day 2, an acute subdural haematoma with midline shift was diagnosed and surgically evacuated on day 3. Despite these life-threatening complications, the patient was discharged to a long-term care facility on day 70 with a Cerebral Performance Category (CPC) score of 3. After rehabilitation, her condition improved to CPC 2, and she survived for several subsequent years.

**Conclusions:**

This case demonstrates that favourable neurological recovery is achievable in elderly adults with hypothermic cardiac arrest, even when complicated by severe haemorrhagic events. The HOPE score is a validated tool for estimating survival probabilities in such cases and may support clinical decision-making regarding extracorporeal life support rewarming.

## Background

Hypothermia is a recognised cause of life-threatening arrhythmias, including ventricular fibrillation, for which extracorporeal cardiopulmonary resuscitation (ECPR) rewarming has emerged as an effective therapeutic approach. The 2021 European Resuscitation Council Guidelines recommended ECPR as the first-line intervention for refractory hypothermic cardiac arrest [1]. Unlike normothermic cardiac arrest, hypothermic cardiac arrest may permit favourable neurological recovery, even in patients aged ≥ 90 years [2] and in those with polytrauma [3].

The Hypothermia Outcome Prediction after Extracorporeal Life Support (HOPE) score is a validated prognostic model that estimates the survival probability at hospital discharge of hypothermic patients in cardiac arrest if rewarmed with extracorporeal life support (ECLS) [4]. Six variables are incorporated, including age, sex, presence of asphyxia, duration of cardiopulmonary resuscitation (CPR), serum potassium concentration, and core temperature at hospital admission [4, 5]. The HOPE-estimated survival probabilities help identify patients most likely to benefit from ECPR and may also predict meaningful neurological recovery, even in elderly adults. A survival probability ≥ 10% has been validated as a pragmatic threshold to guide the avoidance of futile ECPR use [6, 7]. However, the model does not account for in-hospital complications—such as haemorrhage, infection, or neurological deterioration—that may substantially impact outcomes. In elderly patients, additional factors, including frailty, vascular fragility, and multimorbidity, further complicate clinical trajectories.

Here, we describe a rare case of an extremely old patient with hypothermic cardiac arrest who was successfully resuscitated with ECPR and achieved favourable neurological recovery despite severe complications, including cardiac tamponade and intracranial haemorrhage. This case highlights that favourable outcomes remain possible in very elderly adults undergoing ECPR, even in the presence of life-threatening complications.

## Case presentation

A 90-year-old woman with a history of hypertension and mild dementia, previously independent in her activities of daily living, was found collapsed at the entrance of her residence during winter. On arrival, emergency medical services found her moaning and breathing spontaneously, with a palpable carotid pulse but no measurable blood pressure. Her axillary temperature was 21.5 ℃, consistent with severe hypothermia. During transport, she deteriorated into pulseless electrical activity, which subsequently progressed to ventricular fibrillation (VF).

Cardiac arrest persisted upon arrival at the hospital, and the initial cardiac rhythm was asystole. Bladder temperature measurement revealed a core temperature of 22.0 °C. Pupils were 4 mm bilaterally and reactive, and there were no findings suggestive of head trauma. Despite prolonged arrest, initial arterial blood gas analysis showed no evidence of severe metabolic failure: pH 7.33, lactate 2.4 mmol/L, pCO_2_ 49.4 mmHg, HCO_3_⁻ 23.7 mEq/L, and potassium 4.3 mEq/L. Although she was elderly, her pre-arrest functional independence was preserved, so we decided to initiate ECPR for rewarming and circulatory support. VF persisted during the procedure, and the first two consecutive defibrillation attempts at core temperatures in the low 20 ℃ were unsuccessful. Due to technical difficulties, femoral venous access could not be obtained, necessitating cannulation via the right internal jugular vein, which delayed the procedure. Veno-arterial extracorporeal membrane oxygenation (VA-ECMO) was initiated 90 min after hospital arrival (105 min after commencement of CPR), yielding a HOPE-estimated survival probability of 76% [5].

**Fig. 1:**
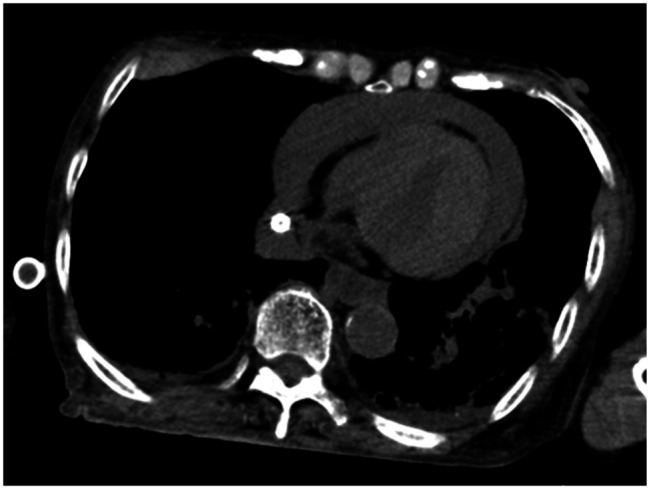
Axial chest computed tomography (CT) image on admission. Imaging revealed a prominent pericardial effusion and fractures of the sternum and multiple ribs, without evidence of pneumothorax or haemothorax

Initial computed tomography (CT) scan revealed no intracranial haemorrhage but demonstrated a pericardial effusion (Fig. 1, 2 A). Pericardiac drainage was performed soon after admission to the intensive care unit (ICU). As cardiac tamponade was suspected to be due to myocardial contusion from prolonged chest compressions or ECMO cannulation, a conservative management strategy without anticoagulants was adopted. Both red blood cells (RBCs) and fresh frozen plasma (FFP) were administered, targeting a fibrinogen level ≥ 1.5 g/L. A total of 500 mL of haematoma was drained within the first hour, necessitating massive transfusion. External rewarming with a MediTherm^®^ device was initiated due to ECMO heat exchanger malfunction, achieving a rewarming rate of approximately 1.5–2.0 °C/h. The core temperature had risen to 30℃ 6 h after arrival, at which point VF converted to sinus rhythm with a single defibrillation. Normothermia (36.0 ℃) was maintained from 8 h after arrival to minimise further bleeding. In total, 1,760 mL of haematoma was drained within 20 h after arrival. Although serum fibrinogen decreased to 1.1 g/L, haemodynamic remained stable with massive transfusion: FFP 2,880 mL (24 units), RBC 2,800 mL (20 units), and platelets 200 mL (10 units) within 24 h (all units according to Japanese standards).

**Fig. 2:**
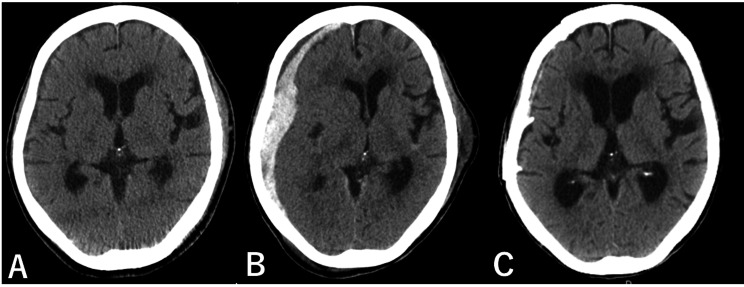
(**A**) Axial head computed tomography (CT) image on admission. No evidence of intracranial haemorrhage was observed at the time of admission. (**A**) Axial head computed tomography (CT) image on day 3 post-admission. A right acute subdural haematoma measuring up to 17 mm in thickness was present, associated with a 6.5 mm midline shift indicative of brain herniation. (**C**) Axial head computed tomography (CT) image on day 67 post-admission. Follow-up axial head CT images on day 67 showed no recurrence of subdural haematoma

As pericardial drainage decreased and haemodynamic stabilised with low-dose catecholamines, ECMO was discontinued 23 h after arrival. Neurological examination on day 3 revealed a Glasgow Coma Scale (GCS) score of E3VNTM5. Head CT showed a right acute subdural haematoma (Fig. 2B). Given the presence of a contralateral subcutaneous haematoma, we diagnosed hypothermia secondary to head trauma sustained during a fall. Emergent craniotomy and haematoma evacuation were performed, with neurological function improving to GCS E4VNTM6 by day 6. She was extubated on day 8 and transferred to a long-term care facility on day 70 with a Cerebral Performance Category (CPC) score of 3 (Fig. 2C). Her GCS was 14 (E4V4M6) at that time. Following rehabilitation, she survived for several years in a wheelchair-dependent state, corresponding to CPC 2. She ultimately died of senility at the age of 94 years.

### Discussion and conclusions

This case represents a rare instance of successful ECPR implementation in a very old adult with severe hypothermic cardiac arrest. Despite life-threatening haemorrhagic complications, including acute subdural haematoma with midline shift and cardiac tamponade, the patient achieved a favourable neurological recovery. This clinical course suggests that profound hypothermia may have exerted a neuroprotective effect, potentially mitigating ischaemic brain injury and contributing to the positive outcome, even in the context of advanced age and multiple critical insults.

Cardiac tamponade in ECPR patients can result from multiple mechanisms, including pre-existing cardiac pathology, prolonged CPR, and ECMO-related procedural complications, such as guidewire manipulation, cannula insertion, and anticoagulation [8, 9]. Furthermore, myocardial injury has been reported in up to 1.7% of cases, especially when resuscitation exceeds 60 min [10, 11]. In our case, myocardial contusion was suspected from either prolonged CPR or internal jugular venous cannulation, exacerbated by coagulopathy. Although substantial pericardial drainage was initially observed, haemodynamic improvement following haematoma evacuation without signs of active pulsatile bleeding suggested myocardial contusion rather than free wall rupture. Consequently, conservative management with transfusion and correction of coagulopathy was prioritised, ultimately achieving haemostasis without surgical intervention.

Acute subdural haematoma requiring surgical evacuation is generally associated with extremely poor outcomes, with reported mortality of approximately 49.3% and unfavourable neurological recovery (Glasgow Outcome Scale 1–3) in 79% of cases, particularly in elderly individuals [12]. Remarkably, our patient developed only mild neurological sequelae, characterised by wheelchair dependence and preservation of baseline cognitive function. Several mechanisms may explain this unexpectedly favourable neurological outcome. First, profound hypothermia may have conferred neuroprotective effects despite the presence of an acute subdural haematoma with midline shift. Hypothermia reduces cerebral metabolic demand and oxygen consumption, thereby limiting ischaemic damage during periods of circulatory arrest [13]. Furthermore, suppression of proinflammatory cytokine release under hypothermic conditions may reduce blood–brain barrier permeability, cerebral oedema, and intracranial pressure (ICP), all of which contribute to attenuating secondary brain injury [14]. Experimental studies have shown that profound hypothermia below 28 °C exerts stronger ICP-lowering and neuroprotective effects than mild-to-moderate hypothermia (30–33 °C), partly through enhanced small ubiquitin-like modifier conjugation that promotes neuronal survival and recovery from ischaemic injury [15]. Although the subdural haematoma was first identified on day 3, earlier intracranial bleeding could not be excluded. The occurrence of cardiac tamponade immediately after ECMO initiation suggests that haemorrhagic complications may have developed earlier during hypothermia or rewarming. The subdural haematoma may therefore have arisen before day 3 but only became radiologically apparent later. Multiple factors—including hypothermia-related coagulopathy [16], rewarming-induced bleeding [17], ECMO-related consumption [18], and myocardial contusion—likely contributed to haematoma expansion and midline shift. Severe hypothermia may nonetheless have mitigated ischaemic brain injury through these neuroprotective effects, thereby contributing to the favourable outcome. Second, minimal CT findings of brain contusion may have limited further neurological deterioration. Moreover, slow rewarming without an ECMO heat exchanger achieved a rate of approximately 1.3 °C/h during the first 6 h. This rate falls within the optimal range (< 5 °C/h) proposed by Saczkowski et al. [19] and avoids the increased mortality associated with excessively slow rewarming (< 0.5 °C/h) in the J-Point registry [20].

Because the survival rate following ECPR decreases with age [21, 22], ECPR is often withheld in elderly individuals, especially the very old. By contrast, the HOPE score was developed and validated using survival to hospital discharge as the primary outcome, and patients with post-rewarming complications were not excluded from the cohort. The HOPE-estimated survival probabilities correspond closely with actual outcomes, even in extreme cases, including paediatric and elderly patients, as previously reported [23]. Our case demonstrated that, even when severe complications develop after rewarming, a high HOPE-estimated survival probability may still be associated with favourable neurological recovery. The HOPE score should therefore not be applied solely in a dichotomous manner but rather interpreted as a continuous probability, balanced against patient-specific factors, such as age or the presence of trauma. High HOPE-estimated survival probabilities may encourage clinicians to pursue post-rewarming treatments and actively manage complications, as meaningful recovery remains achievable, even in the presence of serious post-rewarming events.

Although this case achieved a favourable neurological recovery, the decision to initiate ECPR in extremely old patients cannot be based on the HOPE score alone. Such interventions must be evaluated on a case-by-case basis, taking into account patient characteristics, healthcare resources, and ethical considerations.

In conclusion, this case highlights the potential for neurological recovery in elderly adults with severe hypothermic cardiac arrest, even when complicated by fatal haemorrhagic events. Prognostic tools, such as the HOPE score, may assist decision-making regarding ECLS rewarming in such challenging situations. Still, they must always be considered alongside the patient’s clinical status, individual values, and the availability of healthcare resources.

## Data Availability

All data generated or analysed during this study are included in this published article.

## Abbreviations

CPC: Cerebral Performance Category
CPR: Cardiopulmonary resuscitation
ECPR: Extracorporeal cardiopulmonary resuscitation
ECLS: Extracorporeal Life Support
FFP: Fresh frozen plasma
GCS: Glasgow Coma Scale
HOPE: Hypothermia Outcome Prediction after ECLS
ICP: Intracranial pressure
RBC: Red blood cell
VA-ECMO: Veno-arterial extracorporeal membrane oxygenation
VF: Ventricular fibrillation
